# Impact of Body Mass Index on Survival Outcomes of Patients with Metastatic Renal Cell Carcinoma in the Immuno-oncology Era: A Systematic Review and Meta-analysis

**DOI:** 10.1016/j.euros.2022.03.002

**Published:** 2022-04-04

**Authors:** Kosuke Takemura, Satoru Yonekura, Laura E. Downey, Dimitris Evangelopoulos, Daniel Y.C. Heng

**Affiliations:** a School of Public Health, Imperial College London, London, UK; b Tom Baker Cancer Centre, University of Calgary, Calgary, AB, Canada; c Gustave Roussy Cancer Campus, Villejuif, France

**Keywords:** Body mass index, Immune checkpoint inhibitors, Meta-analysis, Obesity, Prognosis, Renal cell carcinoma

## Abstract

**Context:**

Body mass index (BMI) is a useful tool for measuring body composition. It is unclear whether high BMI is a favourable indicator in patients with metastatic renal cell carcinoma (mRCC) treated with immune checkpoint inhibitors (ICIs).

**Objective:**

To investigate the prognostic significance of BMI in patients with mRCC treated with ICIs in a systematic review and meta-analysis.

**Evidence acquisition:**

Ovid MEDLINE, Embase, and Web of Science were systematically searched in July 2021, and meta-analysis was performed in accordance with the Preferred Reporting Items for Systematic Reviews and Meta-Analyses (PRISMA) statement.

**Evidence synthesis:**

A total of 517 nonduplicate citations were screened by title and abstract, followed by full-text screening of 57 candidate articles to determine whether each study met the eligibility criteria. Overall, a total of 2281 patients from eight studies were included in the systematic review and meta-analysis. BMI levels were compared with overall survival (OS) and progression-free survival (PFS) in seven and three studies, respectively. Overweight/obese BMI was significantly associated with better OS compared to normal BMI (adjusted hazard ratio [aHR] 0.77, 95% confidence intervals [CI] 0.65–0.91; *p* = 0.002). A similar trend was observed for PFS (aHR 0.66, 95% CI 0.44–1.00; *p* = 0.050). There was no statistical heterogeneity or obvious publication bias among these studies.

**Conclusions:**

This is the first systematic review and meta-analysis to evaluate the impact of BMI on survival outcomes of patients with mRCC treated with ICIs. To confirm the existence of the obesity paradox for patients with mRCC in the immuno-oncology era, high-quality clinical trials and basic research are warranted.

**Patient summary:**

We reviewed published data on survival outcomes of 2281 patients with metastatic kidney cancer treated with immunotherapy drugs in relation to their body mass index (BMI). We found that higher BMI was associated with better survival when compared to normal BMI for this disease setting and treatment strategy.

## Take Home Message

High body mass index appears to be associated with lower mortality among patients with metastatic renal cell carcinoma (mRCC) treated with immune checkpoint inhibitors. The role of overweight/obesity in the clinical consequences of mRCC should be extensively investigated in terms of host-related factors and the tumour immune microenvironment.

## 1 Introduction

Excess body weight is an important risk factor for localized renal cell carcinoma (RCC) but is also an established favourable prognostic factor for metastatic RCC (mRCC); this is known as the obesity paradox [1]. Previous meta-analyses demonstrated an interaction between obesity and superior survival outcomes of patients with mRCC, the majority of whom received vascular endothelial growth factor (VEGF) receptor tyrosine kinase inhibitors (TKIs) [2], [3]. Although the mechanisms of this phenomenon are not yet well characterised, one possible explanation is the overexpression of adiponectin receptors such as AdipoR1, which interacts with adipocyte-secreted cytokines and can enhance sunitinib sensitivity via inactivation of the PI3K/AKT/NF-κB signalling pathway, and thus serves as a predictor of the therapeutic effectiveness of VEGF receptor TKIs [4]. However, reports on the obesity paradox for patients treated with immune checkpoint inhibitors (ICIs) are limited, regardless of cancer type, with a few studies conducted in patients with melanoma [5], [6] and non–smallcell lung cancer (NSCLC) [7].

It is known that mRCC is resistant to systemic chemotherapy and thus VEGF receptor TKIs traditionally played a leading role in the treatment of mRCC [8], [9], [10]. Subsequently, several immuno-oncology (IO)-based combination regimens have demonstrated an overall survival (OS) benefit in mRCC, including doublet IO-IO (eg, ipilimumab plus nivolumab) and IO-VEGF strategies (eg, axitinib plus pembrolizumab, cabozantinib plus nivolumab, and lenvatinib plus pembrolizumab). The International mRCC Database Consortium (IMDC) criteria are one of the established prognostic models originally developed in the era of VEGF receptor TKIs that can appropriately stratify patients with mRCC as having favourable, intermediate, or poor risk regarding OS [11], [12]. A more recent study demonstrated that patients were appropriately stratified using the IMDC criteria for OS in the IO-based treatment setting [13]. There is an increasing need for additional reliable risk factors to facilitate risk-directed approaches.

Patients with mRCC frequently have sarcopenia, a condition characterised by degenerative loss of skeletal muscle mass that is an adverse prognostic factor reflecting the degree of cachexia and chronic inflammation [14]. Furthermore, significant improvement in body composition was observed at ≥1 yr from baseline in patients with melanoma, NSCLC, or mRCC treated with ICIs, possibly because of recovery from cancer-related symptoms [15]. Therefore, it can be speculated that body mass index (BMI) may be a prognostic biomarker in patients with mRCC treated with ICIs given that BMI is closely associated with cachexia, chronic inflammation, and subsequent body composition changes. However, the prognostic significance of BMI in patients with mRCC is still controversial, particularly in the era of ICIs [16]. To date, all the systematic reviews of the obesity paradox for patients with mRCC have been conducted regardless of the type/line of systemic treatment (eg, VEGF receptor TKIs), making it uncertain whether BMI retains its prognostic impact in patients with mRCC treated with ICIs only [2], [3], [17], [18].

The aim of the present study was to investigate whether the obesity paradox exists for patients with mRCC treated with ICIs. The specific objectives were to conduct a systematic review and meta-analysis of relevant studies to assess whether high BMI has a positive impact on OS and progression-free survival (PFS) for patients with mRCC treated with ICIs.

## 2 Evidence acquisition

### 2.1 Search strategy

A systematic literature search was performed based on the Preferred Reporting Items for Systematic Reviews and Meta-Analyses (PRISMA) checklist [19]. Peer-reviewed literature, as well as grey literature (eg, abstracts on the American Society of Clinical Oncology Annual Meeting and on the European Society for Medical Oncology Congress) were retrieved from database search of Ovid MEDLINE, Embase, and Web of Science on July 7, 2021. Search terms were divided into three components (BMI, RCC, and ICIs with their variations) to identify eligible studies. The full search strategy developed for each database is provided in the Supplementary material.

### 2.2 Study eligibility

#### 2.2.1 Inclusion criteria

Randomised controlled trials, cohort studies, and case-controlled studies were considered for inclusion. Studies including patients with mRCC or unresectable locally advanced RCC who received ICIs with or without prior systemic treatment were considered for inclusion. Different BMI cutoff values were allowed to enhance the database. Studies with survival outcomes such as the time from ICIs initiation to either death (ie, OS) or disease progression (ie, PFS) were considered for inclusion.

#### 2.2.2 Exclusion criteria

Review articles, case reports, editorial comments, letters, and studies not published in English were excluded. Studies including only patients with non–clear cell RCC were excluded given that the aggressive phenotype of non–clear cell RCC might confound the result of the present study [13]. Studies without comparison by BMI categories (ie, BMI as a continuous variable) were excluded. Studies lacking hazard ratio (HR) estimates, confidence intervals (CI), or sufficient data for accurate calculation were excluded unless unpublished data for such parameters were successfully obtained from the corresponding author.

### 2.3 Study selection

Two investigators (K.T. and S.Y.) screened all titles and abstracts. Full-text screening was conducted if the abstract was not enough to determine whether the study met the inclusion or exclusion criteria. Cohen’s κ coefficient was calculated to assess the level of agreement between the two investigators in the title/abstract and full-text screening processes, after which discrepancy was resolved via discussion. The resulting list of relevant studies was compiled for subsequent quality assessment and meta-analysis, including sensitivity analysis.

### 2.4 Data extraction

The following information was extracted from each selected study: the name of the first author; study period and publication year; study location; cohort size; IMDC risk categories; histological subtypes; systemic treatment regimens; study design; confounders adjusted for; BMI cutoff values; median follow-up; and the HR for survival outcomes with corresponding 95% CI and *p* values. Data were extracted independently by two investigators (K.T. and S.Y.).

### 2.5 Quality assessment

The quality of studies for systematic review was assessed using the Newcastle-Ottawa Scale (NOS), which was developed as an easy and convenient tool for quality assessment of nonrandomised studies in a systematic review [20]. In brief, the NOS has three components: a selection domain (a maximum of four NOS stars for representativeness of the exposed cohort, selection of the nonexposed cohort, ascertainment of exposure, and demonstration that the outcome of interest was not present at the start of the study), a comparability domain (a maximum of two NOS stars for comparability of the cohorts on the basis of the design or analysis controlled for confounders), and an outcome domain (a maximum of three NOS stars for assessment of outcome, follow-up long enough for outcomes to occur, and adequacy of the follow-up for cohorts).

The number of NOS stars was then converted to the trichotomised standard (good, fair, and poor) developed by the Agency for Healthcare Research and Quality (AHRQ) as follows [21]:

• Good quality: three or four NOS stars for the selection domain AND one or two NOS stars for the comparability domain AND two or three NOS stars for the outcome domain.
• Fair quality: two NOS stars for the selection domain AND one or two NOS stars for the comparability domain AND two or three NOS stars for the outcome domain.
• Poor quality: zero or one NOS stars for the selection domain OR zero NOS stars for the comparability domain OR zero or one NOS stars for the outcome domain.

Two investigators (K.T. and S.Y.) performed the quality assessment independently and conflicts were resolved via discussion.

### 2.6 Statistical analyses

Review Manager 5.4.1 (Nordic Cochrane Centre, Copenhagen, Denmark) was utilised for meta-analysis. Associations between BMI and OS/PFS were measured as the HR with corresponding 95% CI and *p* values. HR < 1 suggests superior survival outcomes of patients with high BMI, while HR > 1 indicates inferior survival outcomes. Statistical heterogeneity was evaluated across the studies included using Cochran’s *Q* test and the *I*^2^ statistic under a random-effects model, with a two-sided *p* < 0.10 and *I*^2^ statistic >50% considered statistically significant according to previous guidelines and recommendations [22], [23]. Sensitivity analysis was conducted by removing specific studies from the analysis to examine the influence of their exclusion on the overall estimates. The potential existence of publication bias was visually inspected using funnel plots. Asymmetry tests (eg, the Egger test) were not conducted because they are not recommended for meta-analysis of fewer than ten studies [24]. All statistical analyses were performed with JMP PRO 15.2.0 (SAS Institute, Cary, NC, USA).

## 3 Evidence synthesis

### 3.1 Literature search results

**Fig. 1:**
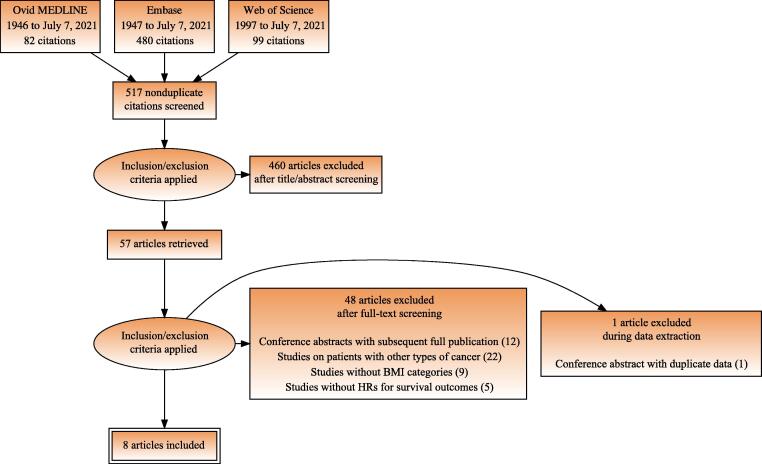
Preferred Reporting Items for Systematic Reviews and Meta-Analyses (PRISMA) diagram depicting the flow of information through the literature search and article selection. BMI = body mass index; HR = hazard ratio.

A flow diagram of the selection process for eligible studies is shown in Figure 1. The search strategy yielded a total of 517 nonduplicate papers, of which 57 articles were considered potentially relevant after all titles and abstracts were screened. Good interobserver agreement was achieved (κ = 0.87). Of the 57 articles, 48 were excluded after full-text screening (12 conference abstracts with subsequent full publication, 22 studies on patients with other types of cancer, nine studies without BMI categories, and five studies with no HR for survival outcomes) and one was excluded during data extraction (a conference abstract with duplicate data), for which substantial interobserver agreement was achieved (κ = 0.79). The remaining eight articles, one of which was a conference abstract, were included in the current systematic review and meta-analysis [25], [26], [27], [28], [29], [30], [31], [32].

### 3.2 Summary of studies

A total of 2281 patients with mRCC treated with ICIs were included for meta-analysis. The baseline characteristics are summarised in Table 1. The proportion of patients who received first-line ICIs varied from 0% to 31% across the studies included. There were two prospective studies (25%) and six retrospective studies (75%). In most studies, BMI was controlled for multiple covariates, including the IMDC criteria, age, and sex, among others. Although there were slight variations of the BMI classification, the cutoff values (18.5, 25, and 30 kg/m^2^) were based on the World Health Organization definitions in all studies. Eight studies (100%) reported the HR for OS and four (50%) reported the HR for PFS as either the crude HR (cHR) or adjusted HR (aHR).

**Table 1:** Baseline characteristics of the studies included in the systematic review

Study	Study period	Cohort, *n*	IMDC risk (%)			Pathology (%)		Therapeutic	Study	Confounders	BMI cutoffs	Median	HR (95% CI)	
	and location	(% FLIO)	FR	IR	PR	CC	NCC	regimens	design	adjusted for	(kg/m^2^)	FU (mo)	OS	PFS
De Giorgi et al, 2019 [25]	2015–2016 Italy	313 (0)	19	70	11	89	9	Nivolumab	PS	Age and SII	≥25 vs <25	>12	cHR 0.67 (0.47–0.95) aHR 0.63 (0.44–0.92)	NA
Labadie et al, 2020 [26]	2011–2018 USA	90 (9)	20	65	6	100	0	Nivolumab, pembrolizumab, or atezolizumab	RS	Brain mets. or irAE a	25–30 vs <25 (≥30 vs <25)	13.5	cHR 0.38 (0.15–0.97)	cHR 0.50 (0.28–0.88)
Sanchez et al, 2020 [27]	2011–2018 USA	203 (24)	18	63	16	100	0	Anti-PD-1/PD-L1 or IO-based combinations	RS	IMDC criteria, age, sex	≥30 vs 18.5–25	>12	cHR 0.54 (0.31–0.95) aHR 0.60 (0.34–1.08)	NA
Colomba et al, 2020 [28]	2016–2018 France	708 (0)	18	56	25	100	0	Nivolumab	PS	IMDC criteria, age, performance status, no. of PLTs	25–30 vs <25 ≥30 vs <25	23.9	cHR 0.82 (0.65–1.03) aHR 0.96 (0.76–1.23)	NA
Martini et al, 2020 [29]	2015–2018 USA	100 (31)	15	55	22	72	20	Anti-PD-1 or IO-based combinations	RS	IMDC criteria, age, sex, race/ethnicity, histology, no. of mets.	≥25 vs <25	>12	aHR 0.51 (0.25–1.02)	aHR 0.61 (0.36–1.04)
Takemura et al, 2020 [30]	2016–2019 Japan	60 (0)	8	85	7	82	18	Nivolumab	RS	Prior nephrectomy and CONUT score	≥25 vs <25	26.4	cHR 0.59 (0.19–1.88) aHR 0.66 (0.19–2.29)	cHR 0.38 (0.15–1.01) aHR 0.60 (0.21–1.69)
Boi et al, 2020 [31]	2015–2019 USA	72 (6)	29	64	6	85	8	Nivolumab or pembrolizumab	RS	IMDC criteria, age, sex, and no. of PLTs	≥25 vs <25 (≥30 vs <30)	>12	cHR 0.90 (0.35–2.32) aHR 0.96 (0.37–2.54)	cHR 0.80 (0.37–1.70) aHR 0.84 (0.39–1.81)
Lalani et al, 2021 [32]	2005–2019 USA	735 (31)	15	51	19	84	15	Anti-PD-1/PD-L1 or IO-based combinations	RS	IMDC criteria, age, sex, race/ethnicity, histology, SFs and type/line of therapy	≥25 vs <25	13.5	cHR 0.58 (0.45–0.75) aHR 0.75 (0.57–0.95)	NA

aHR = adjusted hazard ratio; BMI = body mass index; CC = clear cell; cHR = crude hazard ratio; CI = confidence intervals; CONUT = Controlling Nutritional Status; FLIO = first-line IO; FR = favourable risk; FU = follow-up; HR = hazard ratio; IMDC = International Metastatic Renal Cell Carcinoma Database Consortium; IO = immuno-oncology; irAE = immune-related adverse event; IR = intermediate risk; mets. = metastases; NA = not available; NCC = non–clear cell; OS = overall survival; PFS = progression-free survival; PLTs = prior lines of therapy; PR = poor risk; PS = prospective study; RS = retrospective study; SFs = sarcomatoid features; SII = Systemic Immune-Inflammation Index.

a HR was controlled in a subset of 52 patients with clinical benefit but not in all patients.

### 3.3 Risk of bias within studies

The risk of bias was assessed in accordance with the NOS as listed in Table 2. For the selection domain, six (75%) studies satisfied the requirement for the representativeness item given a multicentre cohort of patients. For the comparability domain, all but one study gained two NOS stars on the basis of multiple confounders involved in the multivariable analysis. For the outcome domain, median follow-up was considered long enough (>12 mo) in all studies. Overall, nine, eight, and seven NOS stars were awarded to two (25%), three (38%), and three (38%) studies, respectively. The methodological quality of all the studies was considered good according to the AHRQ standards.

**Table 2:** Risk of bias assessment according to the NOS and the AHRQ standards

Study	Selection				Comparability	Outcome			Overall quality	
	Representativeness of the exposed cohort a	Selection of the nonexposed cohort	Ascertainment of exposure	Demonstration that outcome of interest was not present at start of study	Comparability of cohorts on the basis of the design or analysis controlled for confounders b	Assessment of outcome	Follow-up long enough for outcomes c	Adequacy of follow-up of cohort	NOS	AHRQ
De Giorgi et al, 2019 [25]	★	★	★	★	★★	★	★	★	9	Good quality
Labadie et al, 2020 [26]	★	★	★	★	★	★	★		7	Good quality
Sanchez et al, 2020 [27]		★	★	★	★★	★	★		7	Good quality
Colomba et al, 2020 [28]	★	★	★	★	★★	★	★		8	Good quality
Martini et al, 2020 [29]		★	★	★	★★	★	★		7	Good quality
Takemura et al, 2020 [30]	★	★	★	★	★★	★	★		8	Good quality
Boi et al, 2020 [31]	★	★	★	★	★★	★	★	★	9	Good quality
Lalani et al, 2021 [32]	★	★	★	★	★★	★	★		8	Good quality

AHRQ = Agency for Healthcare Research and Quality; NOS = Newcastle-Ottawa Scale.

a One NOS star was awarded for a multicentre cohort of patients.

b Two NOS stars were awarded for multiple confounders, while one NOS star was awarded for a single confounder.

c One NOS star was awarded for median follow-up of at least 12 mo.

### 3.4 HR for OS by BMI

**Fig. 2:**
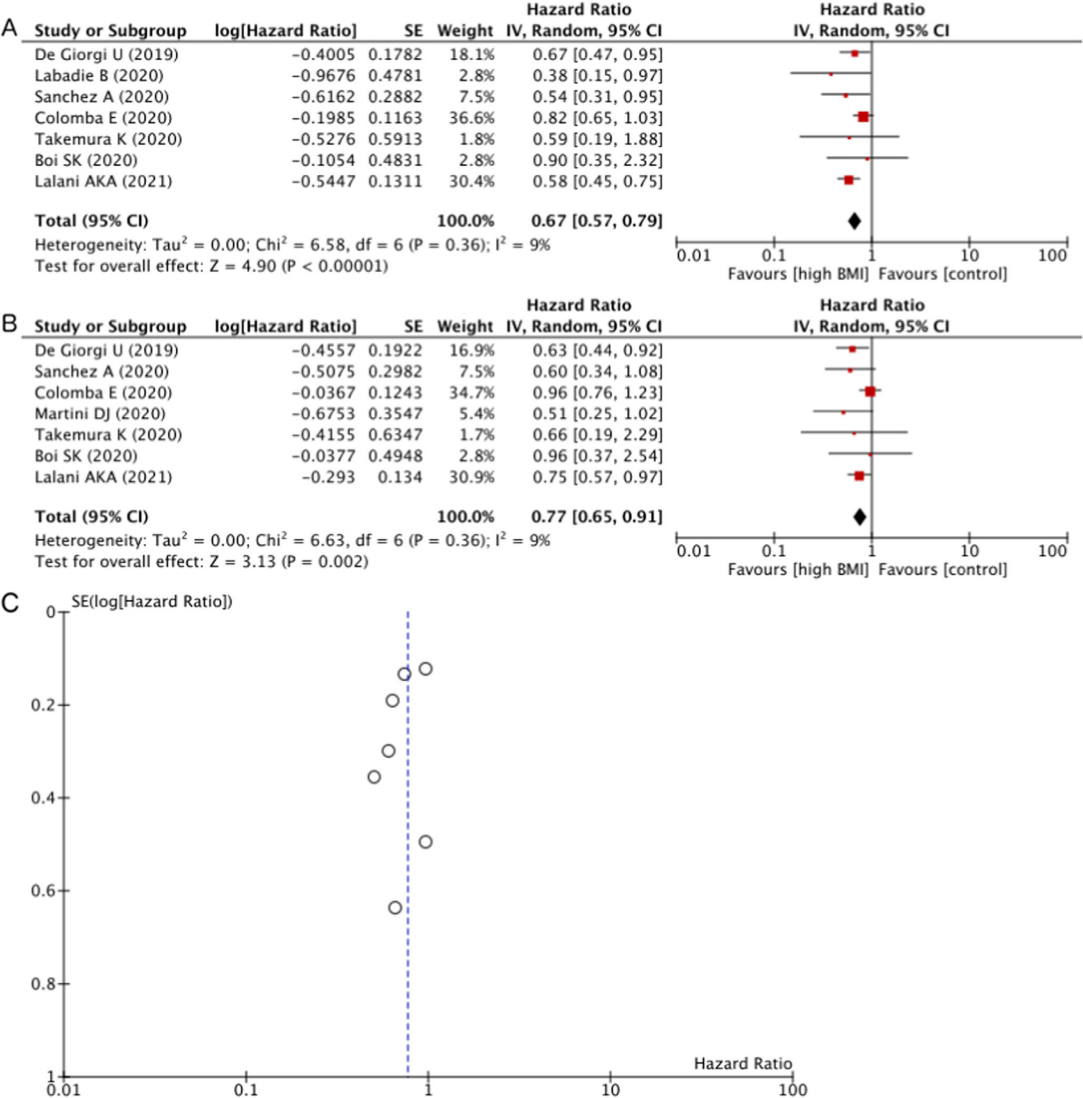
(A) Forest plot of the meta-analysis of the crude HR for OS according to overweight/obese versus normal BMI. (B) Forest plot of the meta-analysis of the adjusted HR for OS according to overweight/obese versus normal BMI controlled for other prognostic factors. (C) Funnel plot of the meta-analysis of the adjusted HR for OS with the effect estimates against their SEs on a reversed scale. BMI = body mass index; CI = confidence intervals; df = degrees of freedom; HR = hazard ratio; IV = inverse variance; OS = overall survival; SE = standard error.

OS was better in the overweight/obese BMI group than in the normal BMI group according to the cHR on univariable analyses (Fig. 2A). Of the seven studies included in the meta-analysis, four (57%) showed significantly better OS for patients with BMI above the normal range according to the cHR. Overall, higher BMI was associated with better OS in comparison to normal BMI (cHR 0.67, 95% CI 0.57–0.79; *p* < 0.001). Neither Cochran’s *Q* test (*P* = 0.360) nor the *I*^2^ statistic (9%) indicated the presence of heterogeneity among these studies. There was one study in which a BMI cutoff of 30 kg/m^2^ (ie, obesity) was used, while all the other studies used a cutoff of 25 kg/m^2^ (ie, overweight) as defined by the World Health Organization [33]. A sensitivity analysis in which the study comparing patients with obese BMI to those with normal BMI was removed similarly demonstrated significantly better OS for patients with overweight BMI than for those with normal BMI (cHR 0.68, 95% CI 0.57–0.81; *p* < 0.001).

To further analyse the effects of covariates that may confound or modify the relationship between BMI and OS, aHR from multivariable analyses was combined (Fig. 2B). Of the seven studies included in the meta-analysis, two (29%) showed significantly better OS for patients with BMI above the normal range according to the aHR. Overall, higher BMI was associated with better OS in comparison to normal BMI, even after adjusting for other risk factors (aHR 0.77, 95% CI 0.65–0.91; *p* = 0.002). Neither Cochran’s *Q* test (*p* = 0.360) nor the *I*^2^ statistic (9%) indicated the presence of heterogeneity among these studies. A funnel plot showed no obvious publication bias regarding OS (Fig. 2C). When the study comparing obese BMI to normal BMI was removed, significantly better OS for patients with overweight BMI remained (aHR 0.78, 95% CI 0.65–0.93; *p* = 0.007).

### 3.5 HR for PFS by BMI

**Fig. 3:**
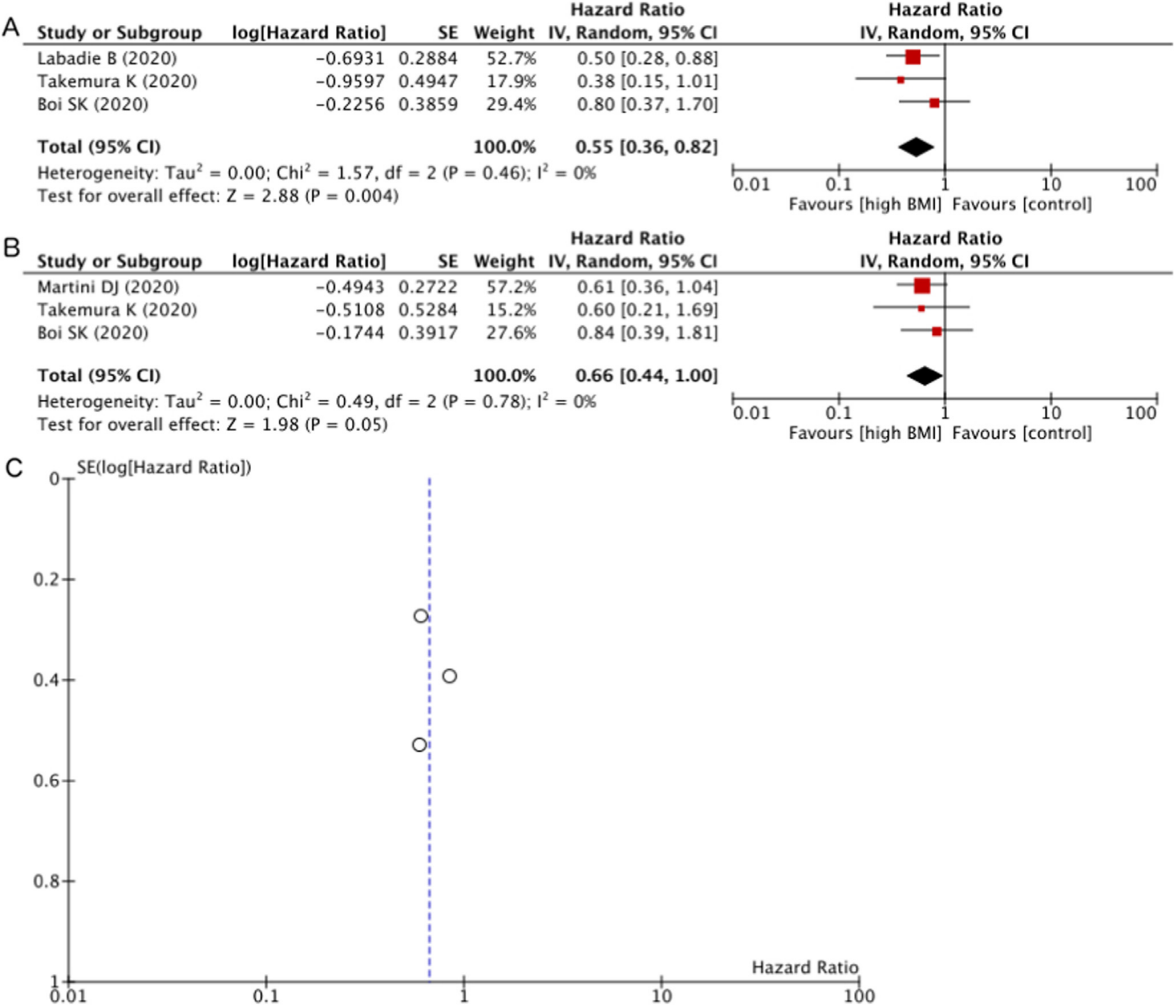
(A) Forest plot of the meta-analysis of the crude HR for PFS according to overweight versus normal BMI. (B) Forest plot of the meta-analysis of the adjusted HR for PFS according to overweight versus normal BMI controlled for other prognostic factors. (C) Funnel plot of the meta-analysis of the adjusted HR for PFS with the effect estimates against their SEs on a reversed scale. BMI = body mass index; CI = confidence intervals; df = degrees of freedom; HR = hazard ratio; IV = inverse variance; PFS = progression-free survival; SE = standard error.

PFS was better for the overweight/obese BMI group than for the normal BMI group according to the cHR on univariable analysis (Fig. 3A). Of the three studies included in the meta-analysis, one (33%) showed significantly better PFS for patients with BMI above the normal range according to the cHR. Overall, higher BMI was significantly associated with better PFS in comparison to normal BMI (cHR 0.55, 95% CI 0.36–0.82; *p* = 0.004). Neither Cochran’s *Q* test (*p* = 0.460) nor the *I*^2^ statistic (0%) indicated the presence of heterogeneity among these studies.

To further analyse the effects of covariates that may confound or modify the relationship between BMI and PFS, aHR from multivariable analyses combined (Fig. 3B). Of the three studies included in the meta-analysis, none (0%) showed significantly better PFS for patients with BMI above the normal range according to the aHR. Overall, overweight BMI showed a trend towards better PFS than normal BMI, even after adjusting for other risk factors (aHR 0.66, 95% CI 0.44–1.00; *p* = 0.050). Neither Cochran’s *Q* test (*p* = 0.780) nor the *I*^2^ statistic (0%) indicated the presence of heterogeneity among these studies. A funnel plot showed no obvious publication bias regarding PFS (Fig. 3C).

### 3.6 Discussion

BMI can easily be calculated from an individual’s body weight and height, and hence is the most basic method for evaluation body composition [34]. Even though it has been reported that high BMI is associated with favourable prognosis for patients with melanoma or NSCLC treated with ICIs [5], [6], [7], it has yet to be fully elucidated whether BMI is a prognostic biomarker in patients with mRCC treated with ICIs [16]. The current systematic review and meta-analysis is, to the best of our knowledge, the first to include patients with mRCC treated with ICIs only. Of note, high BMI was identified as an indicator of favourable prognosis even after adjusting for confounders for patients with mRCC treated with ICIs.

The therapeutic effectiveness of ICIs substantially depends on patient clinical factors and the tumour immune microenvironment [35]. Regarding patient clinical factors, BMI may reflect not only body composition but also physiological and pathological conditions (eg, cachexia and chronic inflammation) relevant to the therapeutic effectiveness of ICIs in patients with mRCC, reflecting their nutritional status [30]. Importantly, a recent report on tumour-bearing mice and patients demonstrated that obesity increased T cells with PD-1 expression and that tumours were more responsive to ICIs in the presence of PD-1-mediated immune suppression induced by obesity [36]. The obesity paradox can therefore be explained in part by favourable host immune status in patients with high BMI, conferring potential survival benefits from ICIs [30].

Regarding the tumour immune microenvironment, adipocytes and infiltrating immune cells release several proinflammatory cytokines and chemokines to establish and maintain local and systemic inflammation, contributing to the consequent suboptimal immune response in overweight/obese individuals [37]. Interestingly, a recent transcriptomic analysis identified several pathways (eg, hypoxia and angiogenesis) that were upregulated in the primary RCC from obese patients in comparison to normal-weight patients, along with canonical inflammatory signatures in the peritumoural adipose tissue in obese patients, which could help to explain the survival advantage conferred by obesity in patients with mRCC receiving systemic treatment [27]. Further exploration of the mechanisms underlying the obesity paradox for patients with mRCC treated with ICIs is required to better understand the complex relationship between BMI and the therapeutic effectiveness of ICIs.

It is of great interest whether active intervention towards weight gain for patients with mRCC treated with ICIs would improve their prognosis or not, which is a possible clinical application of our findings. Indeed, a multimodal supportive care intervention that included physical exercise, nutritional counselling, noninvasive complementary, and alternative medicine, and psychiatric consultation for patients with melanoma treated with ICIs decreased immune-related adverse event rates [38]. Moreover, a randomised controlled trial in patients with NSCLC receiving chemotherapy showed that eicosapentaenoic acid–enriched oral supplementation may have improved nutritional status including low BMI that contributed to numerically better PFS, although the difference was not statistically significant [39]. Furthermore, a multicentre trial demonstrated that patients with melanoma, NSCLC, or mRCC treated with nivolumab whose BMI increased during treatment had significantly better PFS than those whose BMI did not [40]. Given the retrospective nature of this study, the impact of weight gain on the survival advantage from ICIs could not be confirmed, as selection bias was unavoidable. Therefore, prospectively designed and conducted clinical trials are desirable to test the hypothesis that weight gain intervention will improve the prognosis for patients with mRCC treated with ICIs.

There are several limitations of the current systematic review and meta-analysis. First, a relatively small number of studies were included since ICIs were only approved recently. Nonetheless, there were significant associations between BMI and survival outcomes without obvious publication bias. Second, all of the studies except for two were retrospectively conducted. Hence, differences in study design should be properly taken into consideration; however, no statistical heterogeneity was observed across the studies included. High-quality prospective cohort studies should be conducted to verify and validate our findings, even though all of the studies we included were of “good quality” according to the AHRQ standards. Third, the majority of patients had received ICIs as second-line or subsequent systemic treatment. A more contemporary cohort of patients receiving first-line IO-IO or IO-VEGF combinations would yield different results. It has been reported that the relationship between high BMI and favourable prognosis was stronger in patients with melanoma who received first-line ICIs than in those who received non–first-line ICIs [6]. Fourth, confounders involved in the multivariable analysis varied across the studies included. Standardised covariates (eg, the IMDC criteria) for calculation of the BMI's aHR for survival outcomes could provide more accurate information by preventing information bias. Notwithstanding these limitations, the impact of high BMI on superior survival outcomes of patients with mRCC in the IO era appears to be significant.

## 4 Conclusions

The current systematic review and meta-analysis investigated for the first time the existence of the obesity paradox for patients with mRCC treated with ICIs. The prognostic significance of BMI was further confirmed by adjusting for confounders, implying an independent prognostic role of BMI. While there may be a complex interplay between overweight/obesity and the mechanism of action of ICIs, multicentre, large-scale, prospective clinical trials and basic research are required to verify and validate our findings.

  ***Author contributions***: Kosuke Takemura had full access to all the data in the study and takes responsibility for the integrity of the data and the accuracy of the data analysis.

*Study concept and design:* Takemura, Yonekura.

*Acquisition of data:* Takemura, Yonekura, Heng.

*Analysis and interpretation of data:* Takemura, Yonekura, Downey, Evangelopoulos, Heng.

*Drafting of the manuscript:* Takemura.

*Critical revision of the manuscript for important intellectual content:* Downey, Evangelopoulos, Heng.

*Statistical analysis:* Takemura.

*Obtaining funding:* Takemura.

*Administrative, technical, or material support:* Heng.

*Supervision:* None.

*Other:* None.

  ***Financial disclosures:*** Kosuke Takemura certifies that all conflicts of interest, including specific financial interests and relationships and affiliations relevant to the subject matter or materials discussed in the manuscript (eg, employment/affiliation, grants or funding, consultancies, honoraria, stock ownership or options, expert testimony, royalties, or patents filed, received, or pending), are the following: None.

  ***Funding/Support and role of the sponsor*:** This research was supported by the Yasuda Medical Foundation (to K.T.). The sponsor played no direct role in the study.

  ***Acknowledgments*:** The authors thank Dr. Aly-Khan A. Lalani and Ms. Wanling Xie at Dana-Farber Cancer Institute, Dr. Dylan J. Martini at Emory University, and Dr. Lyse A. Norian and Dr. Peng Li at the University of Alabama at Birmingham for their generous provision of unpublished data. In addition, the authors are grateful to Ms. Jacqueline A. Kemp at Imperial College London for her practical advice on search strategy development.

## References

[1] Scelo G., Larose T.L. (2018). Epidemiology and risk factors for kidney cancer. J Clin Oncol.

[2] Bagheri M., Speakman J.R., Shemirani F., Djafarian K. (2016). Renal cell carcinoma survival and body mass index: a dose-response meta-analysis reveals another potential paradox within a paradox. Int J Obes.

[3] Kim L.H., Doan P., He Y., Lau H.M., Pleass H., Patel M.I. (2021). A systematic review and meta-analysis of the significance of body mass index on kidney cancer outcomes. J Urol.

[4] Sun G., Zhang X., Liu Z. (2019). The adiponectin-AdipoR1 axis mediates tumor progression and tyrosine kinase inhibitor resistance in metastatic renal cell carcinoma. Neoplasia.

[5] Naik G.S., Waikar S.S., Johnson A.E.W. (2019). Complex inter-relationship of body mass index, gender and serum creatinine on survival: exploring the obesity paradox in melanoma patients treated with checkpoint inhibition. J Immunother Cancer.

[6] Donnelly D., Bajaj S., Yu J. (2019). The complex relationship between body mass index and response to immune checkpoint inhibition in metastatic melanoma patients. J Immunother Cancer.

[7] Kichenadasse G., Miners J.O., Mangoni A.A., Rowland A., Hopkins A.M., Sorich M.J. (2020). Association between body mass index and overall survival with immune checkpoint inhibitor therapy for advanced non-small cell lung cancer. JAMA Oncol.

[8] Motzer R.J., Hutson T.E., Tomczak P. (2007). Sunitinib versus interferon alfa in metastatic renal-cell carcinoma. N Engl J Med.

[9] Sternberg C.N., Davis I.D., Mardiak J. (2010). Pazopanib in locally advanced or metastatic renal cell carcinoma: results of a randomized phase III trial. J Clin Oncol.

[10] Hudes G., Carducci M., Tomczak P. (2007). Temsirolimus, interferon alfa, or both for advanced renal-cell carcinoma. N Engl J Med.

[11] Heng D.Y., Xie W., Regan M.M. (2009). Prognostic factors for overall survival in patients with metastatic renal cell carcinoma treated with vascular endothelial growth factor-targeted agents: results from a large, multicenter study. J Clin Oncol.

[12] Dudani S., Savard M.F., Heng D.Y.C. (2020). An update on predictive biomarkers in metastatic renal cell carcinoma. Eur Urol Focus.

[13] Yip S.M., Wells C., Moreira R. (2018). Checkpoint inhibitors in patients with metastatic renal cell carcinoma: results from the International Metastatic Renal Cell Carcinoma Database Consortium. Cancer.

[14] Fukushima H., Nakanishi Y., Kataoka M., Tobisu K., Koga F. (2016). Prognostic significance of sarcopenia in patients with metastatic renal cell carcinoma. J Urol.

[15] Patrinely J.R., Young A.C., Quach H. (2020). Survivorship in immune therapy: assessing toxicities, body composition and health-related quality of life among long-term survivors treated with antibodies to programmed death-1 receptor and its ligand. Eur J Cancer.

[16] Gan C.L., Heng D.Y.C. (2020). New insights into the obesity paradox in renal cell carcinoma. Nat Rev Nephrol.

[17] Turco F., Tucci M., Di Stefano R.F. (2021). Renal cell carcinoma (RCC): fatter is better? A review on the role of obesity in RCC. Endocr Relat Cancer.

[18] Aurilio G., Piva F., Santoni M. (2019). The role of obesity in renal cell carcinoma patients: clinical-pathological implications. Int J Mol Sci.

[19] Liberati A., Altman D.G., Tetzlaff J. (2009). The PRISMA statement for reporting systematic reviews and meta-analyses of studies that evaluate healthcare interventions: explanation and elaboration. BMJ.

[20] Deeks J.J., Dinnes J., D’Amico R. (2003). Evaluating non-randomised intervention studies. Health Technol Assess.

[21] Pequeno N.P.F., Cabral N.L.A., Marchioni D.M., Lima S., Lyra C.O. (2020). Quality of life assessment instruments for adults: a systematic review of population-based studies. Health Qual Life Outcomes.

[22] Higgins J., Thompson S., Deeks J., Altman D. (2002). Statistical heterogeneity in systematic reviews of clinical trials: a critical appraisal of guidelines and practice. J Health Serv Res Policy.

[23] Higgins J.P., Thompson S.G., Deeks J.J., Altman D.G. (2003). Measuring inconsistency in meta-analyses. BMJ.

[24] Egger M., Davey Smith G., Schneider M., Minder C. (1997). Bias in meta-analysis detected by a simple, graphical test. BMJ.

[25] De Giorgi U., Procopio G., Giannarelli D. (2019). Association of systemic inflammation index and body mass index with survival in patients with renal cell cancer treated with nivolumab. Clin Cancer Res.

[26] Labadie B.W., Liu P., Bao R. (2019). BMI, irAE, and gene expression signatures associate with resistance to immune-checkpoint inhibition and outcomes in renal cell carcinoma. J Transl Med.

[27] Sanchez A., Furberg H., Kuo F. (2020). Transcriptomic signatures related to the obesity paradox in patients with clear cell renal cell carcinoma: a cohort study. Lancet Oncol.

[28] Colomba E., Dalban C., Flechon A. (2020). Is body mass index (BMI) associated with favorable outcomes in metastatic renal cell carcinoma (mRCC) treated with nivolumab? An ancillary study of the NIVOREN-GETUG AFU-26 trial. J Clin Oncol.

[29] Martini D.J., Liu Y., Shabto J.M. (2020). Novel risk scoring system for patients with metastatic renal cell carcinoma treated with immune checkpoint inhibitors. Oncologist.

[30] Takemura K., Yuasa T., Fujiwara R. (2020). Prognostic Significance of the Controlling Nutritional Status (CONUT) score in patients with advanced renal cell carcinoma treated with nivolumab after failure of prior tyrosine kinase inhibitors. J Urol.

[31] Boi S.K., Orlandella R.M., Gibson J.T. (2020). Obesity diminishes response to PD-1-based immunotherapies in renal cancer. J Immunother Cancer.

[32] Lalani A.A., Bakouny Z., Farah S. (2021). Assessment of immune checkpoint inhibitors and genomic alterations by body mass index in advanced renal cell carcinoma. JAMA Oncol.

[33] World Health Organization (1995). Physical status: the use and interpretation of anthropometry. Report of a WHO Expert Committee. World Health Organ Tech Rep Ser.

[34] Liu P., Ma F., Lou H., Liu Y. (2013). The utility of fat mass index vs. body mass index and percentage of body fat in the screening of metabolic syndrome. BMC Public Health.

[35] Yuasa T., Masuda H., Yamamoto S., Numao N., Yonese J. (2017). Biomarkers to predict prognosis and response to checkpoint inhibitors. Int J Clin Oncol.

[36] Wang Z., Aguilar E.G., Luna J.I. (2019). Paradoxical effects of obesity on T cell function during tumor progression and PD-1 checkpoint blockade. Nat Med.

[37] Frasca D., Diaz A., Romero M., Thaller S., Blomberg B.B. (2018). Secretion of autoimmune antibodies in the human subcutaneous adipose tissue. PLoS One.

[38] Lacey J., Lomax A.J., McNeil C. (2019). A supportive care intervention for people with metastatic melanoma being treated with immunotherapy: a pilot study assessing feasibility, perceived benefit, and acceptability. Support Care Cancer.

[39] Sanchez-Lara K., Turcott J.G., Juarez-Hernandez E. (2014). Effects of an oral nutritional supplement containing eicosapentaenoic acid on nutritional and clinical outcomes in patients with advanced non-small cell lung cancer: randomised trial. Clin Nutr.

[40] Nitipir C., Orlov-Slavu C., Alecu L. (2020). Possible influence of weight gain and creatinine levels in predicting response to nivolumab: a multicenter analysis. Metabolites.

