# Supplementary material for: Impact of Body Mass Index on Survival Outcomes of Patients with Metastatic Renal Cell Carcinoma in the Immuno-oncology Era: A Systematic Review and Meta-analysis
Source: Eur Urol Open Sci. 2022 Apr 4;39:62–71. doi: 10.1016/j.euros.2022.03.002 (PMC9068728; doi:10.1016/j.euros.2022.03.002)
Supplement: Supplementary Data 1 [file mmc1.docx]

**Supplementary material**

**Ovid MEDLINE search strategy**

1. exp Body Mass Index/

2. exp Obesity/

3. exp Overweight/

4. (body mass index or body weight or cachexia or nutrition* or obese or obesity or overweight or BMI).mp.

5. 1 or 2 or 3 or 4

6. exp Carcinoma, Renal Cell/

7. exp Kidney Neoplasms/

8. (((kidney or renal) adj2 (adenocarcinoma* or cancer* or carcinoma* or malignan* or neoplas* or tumo?r*)) or ?RCC).mp.

9. 6 or 7 or 8

10. exp Immunotherapy/

11. ((checkpoint and (blockade or inhibit*)) or immunotherap* or (programmed adj2 death) or ICB? or ICI? or PD-?1).mp.

12. (atezolizumab or avelumab or durvalumab or ipilimumab or nivolumab or pembrolizumab).mp.

13. 10 or 11 or 12

14. 5 and 9 and 13

**Embase search strategy**

1. exp body mass/

2. exp body weight/

3. exp obesity/

4. (body mass index or body weight or cachexia or nutrition* or obese or obesity or overweight or BMI).mp.

5. 1 or 2 or 3 or 4

6. exp kidney carcinoma/

7. exp kidney tumor/

8. (((kidney or renal) adj2 (adenocarcinoma* or cancer* or carcinoma* or malignan* or neoplas* or tumo?r*)) or ?RCC).mp.

9. 6 or 7 or 8

10. exp immunotherapy/

11. ((checkpoint and (blockade or inhibit*)) or immunotherap* or (programmed adj2 death) or ICB? or ICI? or PD-?1).mp.

12. (atezolizumab or avelumab or durvalumab or ipilimumab or nivolumab or pembrolizumab).mp.

13. 10 or 11 or 12

14. 5 and 9 and 13

**Web of Science search strategy**

1. body mass index or body weight or cachexia or nutrition* or obese or obesity or overweight or BMI

2. (kidney or renal) near/1 (adenocarcinoma* or cancer* or carcinoma* or malignan* or neoplas* or tumo?r*) or ?RCC

3. checkpoint and (blockade or inhibit*) or immunotherap* or (programmed near/1 death) or ICB? or ICI? or PD-?1 or atezolizumab or avelumab or durvalumab or ipilimumab or nivolumab or pembrolizumab

4. 1 and 2 and 3
